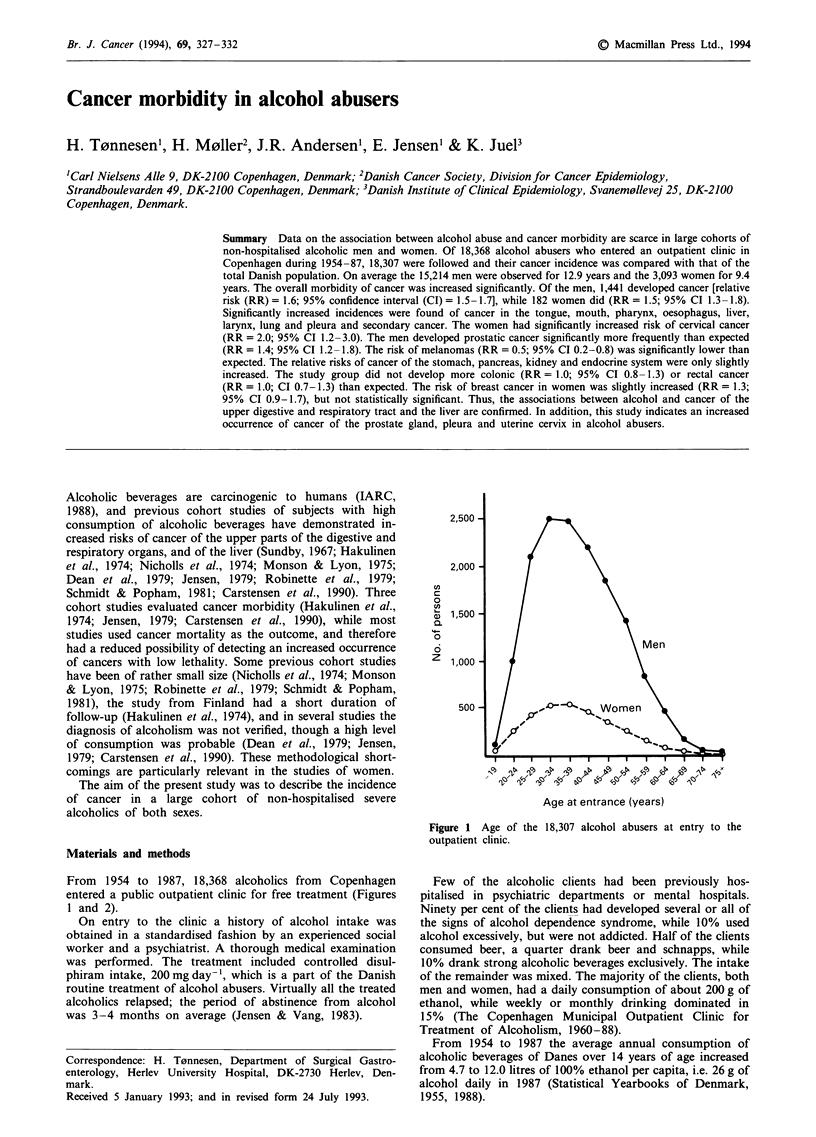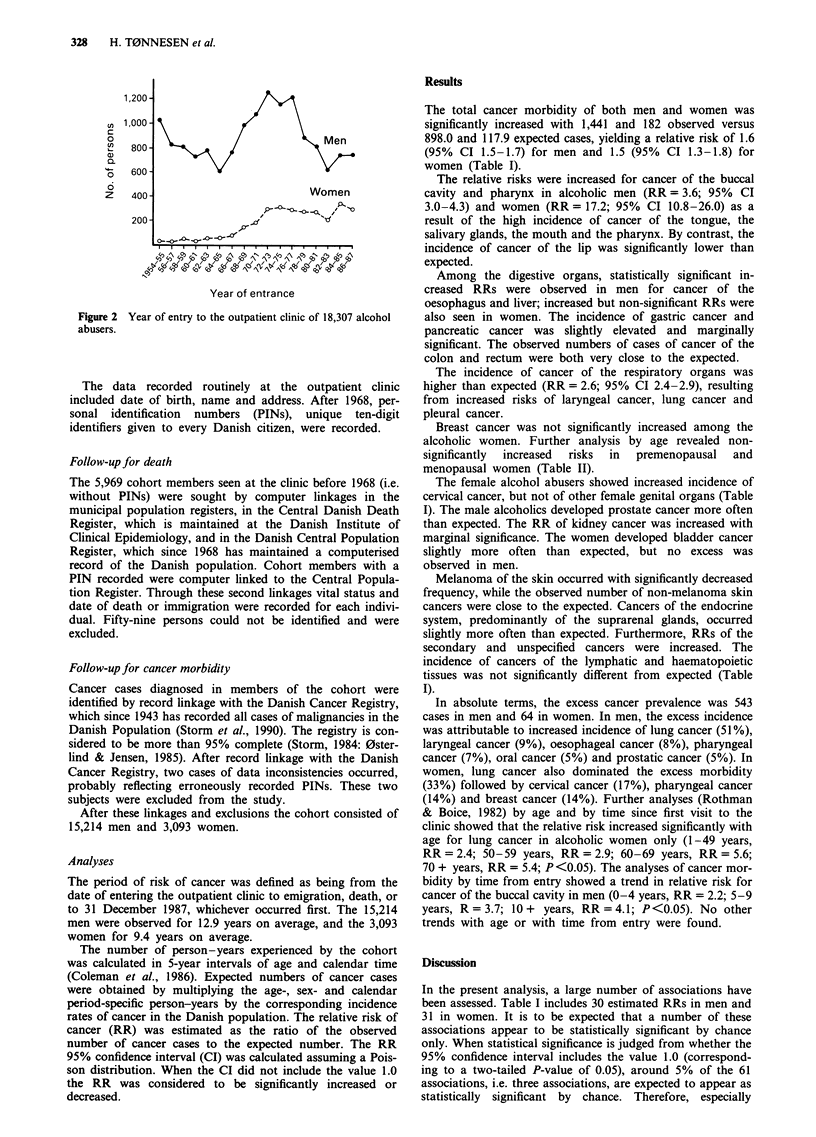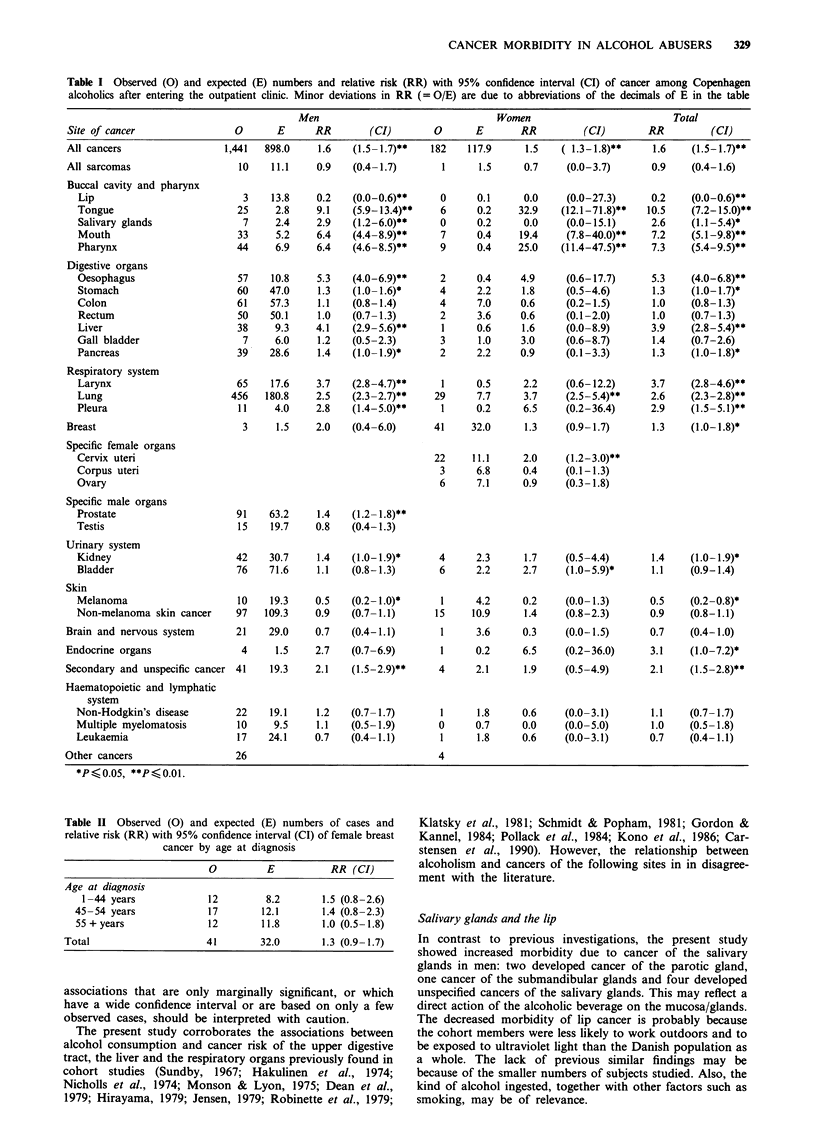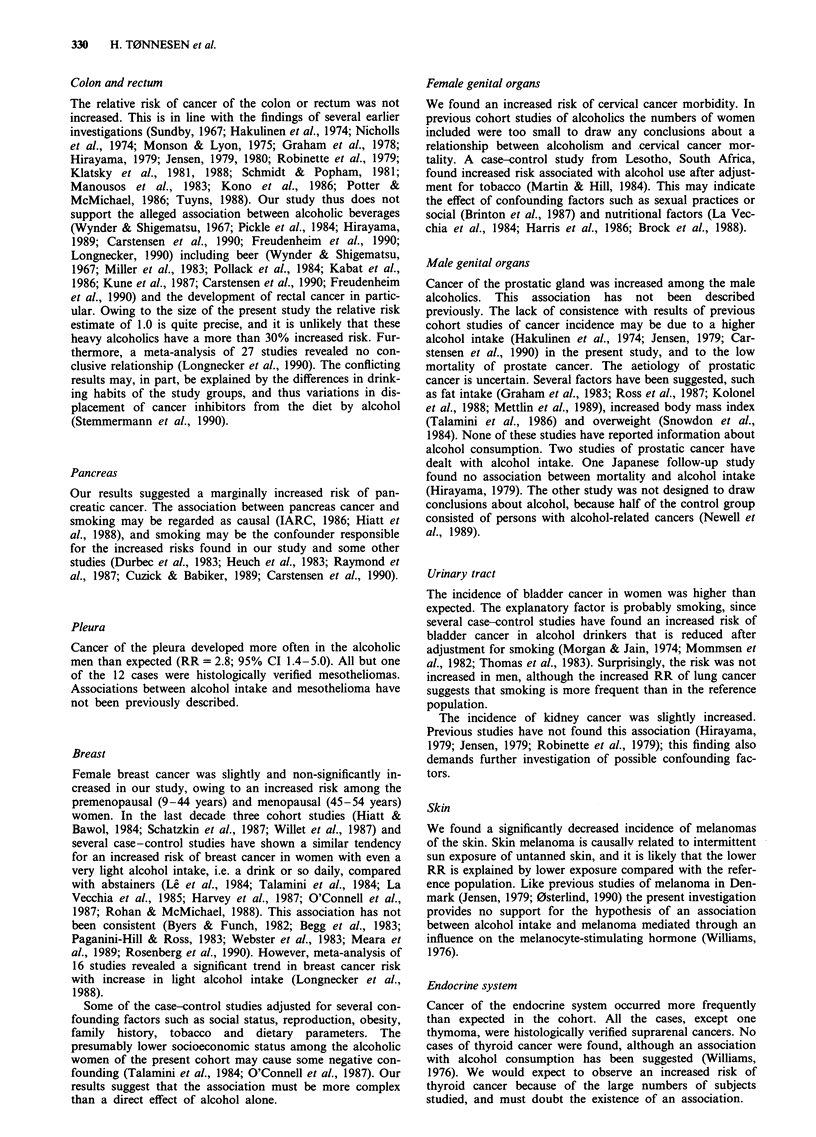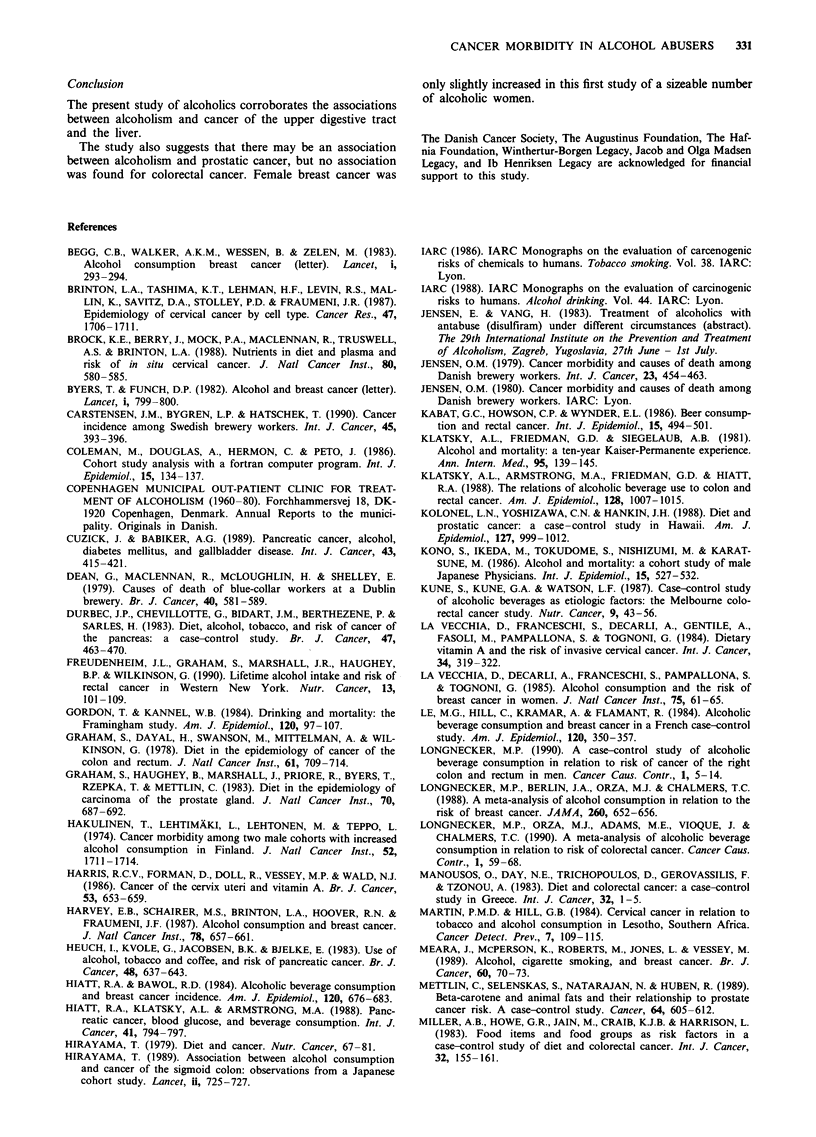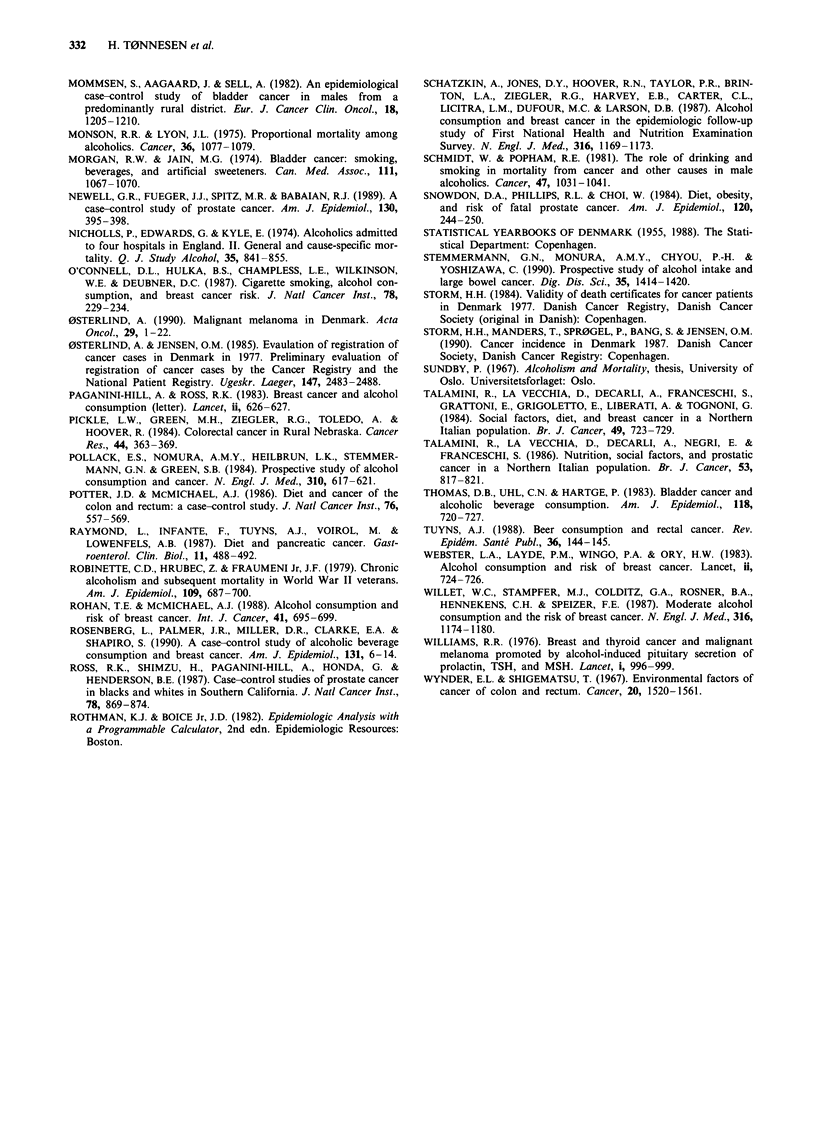# Cancer morbidity in alcohol abusers.

**DOI:** 10.1038/bjc.1994.59

**Published:** 1994-02

**Authors:** H. Tønnesen, H. Møller, J. R. Andersen, E. Jensen, K. Juel

**Affiliations:** Carl Nielsens Alle 9, Copenhagen, Denmark.

## Abstract

Data on the association between alcohol abuse and cancer morbidity are scarce in large cohorts of non-hospitalised alcoholic men and women. Of 18,368 alcohol abusers who entered an outpatient clinic in Copenhagen during 1954-87, 18,307 were followed and their cancer incidence was compared with that of the total Danish population. On average the 15,214 men were observed for 12.9 years and the 3,093 women for 9.4 years. The overall morbidity of cancer was increased significantly. Of the men, 1,441 developed cancer [relative risk (RR) = 1.6; 95% confidence interval (CI) = 1.5-1.7], while 182 women did (RR = 1.5; 95% CI 1.3-1.8). Significantly increased incidences were found of cancer in the tongue, mouth, pharynx, oesophagus, liver, larynx, lung and pleura and secondary cancer. The women had significantly increased risk of cervical cancer (RR = 2.0; 95% CI 1.2-3.0). The men developed prostatic cancer significantly more frequently than expected (RR = 1.4; 95% CI 1.2-1.8). The risk of melanomas (RR = 0.5; 95% CI 0.2-0.8) was significantly lower than expected. The relative risks of cancer of the stomach, pancreas, kidney and endocrine system were only slightly increased. The study group did not develop more colonic (RR = 1.0; 95% CI 0.8-1.3) or rectal cancer (RR = 1.0; CI 0.7-1.3) than expected. The risk of breast cancer in women was slightly increased (RR = 1.3; 95% CI 0.9-1.7), but not statistically significant. Thus, the associations between alcohol and cancer of the upper digestive and respiratory tract and the liver are confirmed. In addition, this study indicates an increased occurrence of cancer of the prostate gland, pleura and uterine cervix in alcohol abusers.


Br. J. Cancer (1994), 69, 327-332 © Macmillan Press Ltd., 1994

### Cancer morbidity in alcohol abusers

H. Tønnesen[1], H. Møller[2], J.R. Andersen[1], E. Jensen[1] & K. Juel[3]

[1]*Carl Nielsens Alle 9, DK-2100 Copenhagen, Denmark;* [2]*Danish Cancer Society, Division for Cancer Epidemiology, Strandboulevarden 49, DK-2100 Copenhagen, Denmark;* [3]*Danish Institute of Clinical Epidemiology, Svanemøllevej 25, DK-2100 Copenhagen, Denmark.*

**Summary** Data on the association between alcohol abuse and cancer morbidity are scarce in large cohorts of non-hospitalised alcoholic men and women. Of 18,368 alcohol abusers who entered an outpatient clinic in Copenhagen during 1954-87, 18,307 were followed and their cancer incidence was compared with that of the total Danish population. On average the 15,214 men were observed for 12.9 years and the 3,093 women for 9.4 years. The overall morbidity of cancer was increased significantly. Of the men, 1,441 developed cancer [relative risk (RR) = 1.6; 95% confidence interval (CI) = 1.5-1.7], while 182 women did (RR = 1.5; 95% CI 1.3-1.8). Significantly increased incidences were found of cancer in the tongue, mouth, pharynx, oesophagus, liver, larynx, lung and pleura and secondary cancer. The women had significantly increased risk of cervical cancer (RR = 2.0; 95% CI 1.2-3.0). The men developed prostatic cancer significantly more frequently than expected (RR = 1.4; 95% CI 1.2-1.8). The risk of melanomas (RR = 0.5; 95% CI 0.2-0.8) was significantly lower than expected. The relative risks of cancer of the stomach, pancreas, kidney and endocrine system were only slightly increased. The study group did not develop more colonic (RR = 1.0; 95% CI 0.8-1.3) or rectal cancer (RR = 1.0; CI 0.7-1.3) than expected. The risk of breast cancer in women was slightly increased (RR = 1.3; 95% CI 0.9-1.7), but not statistically significant. Thus, the associations between alcohol and cancer of the upper digestive and respiratory tract and the liver are confirmed. In addition, this study indicates an increased occurrence of cancer of the prostate gland, pleura and uterine cervix in alcohol abusers.

Alcoholic beverages are carcinogenic to humans (IARC, 1988), and previous cohort studies of subjects with high consumption of alcoholic beverages have demonstrated increased risks of cancer of the upper parts of the digestive and respiratory organs, and of the liver (Sundby, 1967; Hakulinen *et al.*, 1974; Nicholls *et al.*, 1974; Monson & Lyon, 1975; Dean *et al.*, 1979; Jensen, 1979; Robinette *et al.*, 1979; Schmidt & Popham, 1981; Carstensen *et al.*, 1990). Three cohort studies evaluated cancer morbidity (Hakulinen *et al.*, 1974; Jensen, 1979; Carstensen *et al.*, 1990), while most studies used cancer mortality as the outcome, and therefore had a reduced possibility of detecting an increased occurrence of cancers with low lethality. Some previous cohort studies have been of rather small size (Nicholls *et al.*, 1974; Monson & Lyon, 1975; Robinette *et al.*, 1979; Schmidt & Popham, 1981), the study from Finland had a short duration of follow-up (Hakulinen *et al.*, 1974), and in several studies the diagnosis of alcoholism was not verified, though a high level of consumption was probable (Dean *et al.*, 1979; Jensen, 1979; Carstensen *et al.*, 1990). These methodological shortcomings are particularly relevant in the studies of women.

The aim of the present study was to describe the incidence of cancer in a large cohort of non-hospitalised severe alcoholics of both sexes.

### Materials and methods

From 1954 to 1987, 18,368 alcoholics from Copenhagen entered a public outpatient clinic for free treatment (Figures 1 and 2).

On entry to the clinic a history of alcohol intake was obtained in a standardised fashion by an experienced social worker and a psychiatrist. A thorough medical examination was performed. The treatment included controlled disulphiram intake, 200 mg day$^{-1}$, which is a part of the Danish routine treatment of alcohol abusers. Virtually all the treated alcoholics relapsed; the period of abstinence from alcohol was 3-4 months on average (Jensen & Vang, 1983).

Correspondence: H. Tønnesen, Department of Surgical Gastroenterology, Herlev University Hospital, DK-2730 Herlev, Denmark.
Received 5 January 1993; and in revised form 24 July 1993.

**Figure 1** Age of the 18,307 alcohol abusers at entry to the outpatient clinic.

Few of the alcoholic clients had been previously hospitalised in psychiatric departments or mental hospitals. Ninety per cent of the clients had developed several or all of the signs of alcohol dependence syndrome, while 10% used alcohol excessively, but were not addicted. Half of the clients consumed beer, a quarter drank beer and schnapps, while 10% drank strong alcoholic beverages exclusively. The intake of the remainder was mixed. The majority of the clients, both men and women, had a daily consumption of about 200 g of ethanol, while weekly or monthly drinking dominated in 15% (The Copenhagen Municipal Outpatient Clinic for Treatment of Alcoholism, 1960-88).

From 1954 to 1987 the average annual consumption of alcoholic beverages of Danes over 14 years of age increased from 4.7 to 12.0 litres of 100% ethanol per capita, i.e. 26 g of alcohol daily in 1987 (Statistical Yearbooks of Denmark, 1955, 1988).

**Figure 2** Year of entry to the outpatient clinic of 18,307 alcohol abusers.

The data recorded routinely at the outpatient clinic included date of birth, name and address. After 1968, personal identification numbers (PINs), unique ten-digit identifiers given to every Danish citizen, were recorded.

*Follow-up for death*

The 5,969 cohort members seen at the clinic before 1968 (i.e. without PINs) were sought by computer linkages in the municipal population registers, in the Central Danish Death Register, which is maintained at the Danish Institute of Clinical Epidemiology, and in the Danish Central Population Register, which since 1968 has maintained a computerised record of the Danish population. Cohort members with a PIN recorded were computer linked to the Central Population Register. Through these second linkages vital status and date of death or immigration were recorded for each individual. Fifty-nine persons could not be identified and were excluded.

*Follow-up for cancer morbidity*

Cancer cases diagnosed in members of the cohort were identified by record linkage with the Danish Cancer Registry, which since 1943 has recorded all cases of malignancies in the Danish Population (Storm *et al.*, 1990). The registry is considered to be more than 95% complete (Storm, 1984: Østerlind & Jensen, 1985). After record linkage with the Danish Cancer Registry, two cases of data inconsistencies occurred, probably reflecting erroneously recorded PINs. These two subjects were excluded from the study.

After these linkages and exclusions the cohort consisted of 15,214 men and 3,093 women.

*Analyses*

The period of risk of cancer was defined as being from the date of entering the outpatient clinic to emigration, death, or to 31 December 1987, whichever occurred first. The 15,214 men were observed for 12.9 years on average, and the 3,093 women for 9.4 years on average.

The number of person–years experienced by the cohort was calculated in 5-year intervals of age and calendar time (Coleman *et al.*, 1986). Expected numbers of cancer cases were obtained by multiplying the age-, sex- and calendar period-specific person–years by the corresponding incidence rates of cancer in the Danish population. The relative risk of cancer (RR) was estimated as the ratio of the observed number of cancer cases to the expected number. The RR 95% confidence interval (CI) was calculated assuming a Poisson distribution. When the CI did not include the value 1.0 the RR was considered to be significantly increased or decreased.

### Results

The total cancer morbidity of both men and women was significantly increased with 1,441 and 182 observed versus 898.0 and 117.9 expected cases, yielding a relative risk of 1.6 (95% CI 1.5–1.7) for men and 1.5 (95% CI 1.3–1.8) for women (Table I).

The relative risks were increased for cancer of the buccal cavity and pharynx in alcoholic men (RR = 3.6; 95% CI 3.0–4.3) and women (RR = 17.2; 95% CI 10.8–26.0) as a result of the high incidence of cancer of the tongue, the salivary glands, the mouth and the pharynx. By contrast, the incidence of cancer of the lip was significantly lower than expected.

Among the digestive organs, statistically significant increased RRs were observed in men for cancer of the oesophagus and liver; increased but non-significant RRs were also seen in women. The incidence of gastric cancer and pancreatic cancer was slightly elevated and marginally significant. The observed numbers of cases of cancer of the colon and rectum were both very close to the expected.

The incidence of cancer of the respiratory organs was higher than expected (RR = 2.6; 95% CI 2.4–2.9), resulting from increased risks of laryngeal cancer, lung cancer and pleural cancer.

Breast cancer was not significantly increased among the alcoholic women. Further analysis by age revealed non-significantly increased risks in premenopausal and menopausal women (Table II).

The female alcohol abusers showed increased incidence of cervical cancer, but not of other female genital organs (Table I). The male alcoholics developed prostate cancer more often than expected. The RR of kidney cancer was increased with marginal significance. The women developed bladder cancer slightly more often than expected, but no excess was observed in men.

Melanoma of the skin occurred with significantly decreased frequency, while the observed number of non-melanoma skin cancers were close to the expected. Cancers of the endocrine system, predominantly of the suprarenal glands, occurred slightly more often than expected. Furthermore, RRs of the secondary and unspecified cancers were increased. The incidence of cancers of the lymphatic and haematopoietic tissues was not significantly different from expected (Table I).

In absolute terms, the excess cancer prevalence was 543 cases in men and 64 in women. In men, the excess incidence was attributable to increased incidence of lung cancer (51%), laryngeal cancer (9%), oesophageal cancer (8%), pharyngeal cancer (7%), oral cancer (5%) and prostatic cancer (5%). In women, lung cancer also dominated the excess morbidity (33%) followed by cervical cancer (17%), pharyngeal cancer (14%) and breast cancer (14%). Further analyses (Rothman & Boice, 1982) by age and by time since first visit to the clinic showed that the relative risk increased significantly with age for lung cancer in alcoholic women only (1–49 years, RR = 2.4; 50–59 years, RR = 2.9; 60–69 years, RR = 5.6; 70 + years, RR = 5.4; $P < 0.05$). The analyses of cancer morbidity by time from entry showed a trend in relative risk for cancer of the buccal cavity in men (0–4 years, RR = 2.2; 5–9 years, R = 3.7; 10 + years, RR = 4.1; $P < 0.05$). No other trends with age or with time from entry were found.

### Discussion

In the present analysis, a large number of associations have been assessed. Table I includes 30 estimated RRs in men and 31 in women. It is to be expected that a number of these associations appear to be statistically significant by chance only. When statistical significance is judged from whether the 95% confidence interval includes the value 1.0 (corresponding to a two-tailed $P$-value of 0.05), around 5% of the 61 associations, i.e. three associations, are expected to appear as statistically significant by chance. Therefore, especially

**Table I** Observed (O) and expected (E) numbers and relative risk (RR) with 95% confidence interval (CI) of cancer among Copenhagen alcoholics after entering the outpatient clinic. Minor deviations in RR (= O/E) are due to abbreviations of the decimals of E in the table

| | *Men* | | | | *Women* | | | | *Total* | |
|---|---|---|---|---|---|---|---|---|---|---|
| *Site of cancer* | *O* | *E* | *RR* | *(CI)* | *O* | *E* | *RR* | *(CI)* | *RR* | *(CI)* |
| All cancers | 1,441 | 898.0 | 1.6 | (1.5–1.7)** | 182 | 117.9 | 1.5 | ( 1.3–1.8)** | 1.6 | (1.5–1.7)** |
| All sarcomas | 10 | 11.1 | 0.9 | (0.4–1.7) | 1 | 1.5 | 0.7 | (0.0–3.7) | 0.9 | (0.4–1.6) |
| Buccal cavity and pharynx | | | | | | | | | | |
| Lip | 3 | 13.8 | 0.2 | (0.0–0.6)** | 0 | 0.1 | 0.0 | (0.0–27.3) | 0.2 | (0.0–0.6)** |
| Tongue | 25 | 2.8 | 9.1 | (5.9–13.4)** | 6 | 0.2 | 32.9 | (12.1–71.8)** | 10.5 | (7.2–15.0)** |
| Salivary glands | 7 | 2.4 | 2.9 | (1.2–6.0)** | 0 | 0.2 | 0.0 | (0.0–15.1) | 2.6 | (1.1–5.4)* |
| Mouth | 33 | 5.2 | 6.4 | (4.4–8.9)** | 7 | 0.4 | 19.4 | (7.8–40.0)** | 7.2 | (5.1–9.8)** |
| Pharynx | 44 | 6.9 | 6.4 | (4.6–8.5)** | 9 | 0.4 | 25.0 | (11.4–47.5)** | 7.3 | (5.4–9.5)** |
| Digestive organs | | | | | | | | | | |
| Oesophagus | 57 | 10.8 | 5.3 | (4.0–6.9)** | 2 | 0.4 | 4.9 | (0.6–17.7) | 5.3 | (4.0–6.8)** |
| Stomach | 60 | 47.0 | 1.3 | (1.0–1.6)* | 4 | 2.2 | 1.8 | (0.5–4.6) | 1.3 | (1.0–1.7)* |
| Colon | 61 | 57.3 | 1.1 | (0.8–1.4) | 4 | 7.0 | 0.6 | (0.2–1.5) | 1.0 | (0.8–1.3) |
| Rectum | 50 | 50.1 | 1.0 | (0.7–1.3) | 2 | 3.6 | 0.6 | (0.1–2.0) | 1.0 | (0.7–1.3) |
| Liver | 38 | 9.3 | 4.1 | (2.9–5.6)** | 1 | 0.6 | 1.6 | (0.0–8.9) | 3.9 | (2.8–5.4)** |
| Gall bladder | 7 | 6.0 | 1.2 | (0.5–2.3) | 3 | 1.0 | 3.0 | (0.6–8.7) | 1.4 | (0.7–2.6) |
| Pancreas | 39 | 28.6 | 1.4 | (1.0–1.9)* | 2 | 2.2 | 0.9 | (0.1–3.3) | 1.3 | (1.0–1.8)* |
| Respiratory system | | | | | | | | | | |
| Larynx | 65 | 17.6 | 3.7 | (2.8–4.7)** | 1 | 0.5 | 2.2 | (0.6–12.2) | 3.7 | (2.8–4.6)** |
| Lung | 456 | 180.8 | 2.5 | (2.3–2.7)** | 29 | 7.7 | 3.7 | (2.5–5.4)** | 2.6 | (2.3–2.8)** |
| Pleura | 11 | 4.0 | 2.8 | (1.4–5.0)** | 1 | 0.2 | 6.5 | (0.2–36.4) | 2.9 | (1.5–5.1)** |
| Breast | 3 | 1.5 | 2.0 | (0.4–6.0) | 41 | 32.0 | 1.3 | (0.9–1.7) | 1.3 | (1.0–1.8)* |
| Specific female organs | | | | | | | | | | |
| Cervix uteri | | | | | 22 | 11.1 | 2.0 | (1.2–3.0)** | | |
| Corpus uteri | | | | | 3 | 6.8 | 0.4 | (0.1–1.3) | | |
| Ovary | | | | | 6 | 7.1 | 0.9 | (0.3–1.8) | | |
| Specific male organs | | | | | | | | | | |
| Prostate | 91 | 63.2 | 1.4 | (1.2–1.8)** | | | | | | |
| Testis | 15 | 19.7 | 0.8 | (0.4–1.3) | | | | | | |
| Urinary system | | | | | | | | | | |
| Kidney | 42 | 30.7 | 1.4 | (1.0–1.9)* | 4 | 2.3 | 1.7 | (0.5–4.4) | 1.4 | (1.0–1.9)* |
| Bladder | 76 | 71.6 | 1.1 | (0.8–1.3) | 6 | 2.2 | 2.7 | (1.0–5.9)* | 1.1 | (0.9–1.4) |
| Skin | | | | | | | | | | |
| Melanoma | 10 | 19.3 | 0.5 | (0.2–1.0)* | 1 | 4.2 | 0.2 | (0.0–1.3) | 0.5 | (0.2–0.8)* |
| Non-melanoma skin cancer | 97 | 109.3 | 0.9 | (0.7–1.1) | 15 | 10.9 | 1.4 | (0.8–2.3) | 0.9 | (0.8–1.1) |
| Brain and nervous system | 21 | 29.0 | 0.7 | (0.4–1.1) | 1 | 3.6 | 0.3 | (0.0–1.5) | 0.7 | (0.4–1.0) |
| Endocrine organs | 4 | 1.5 | 2.7 | (0.7–6.9) | 1 | 0.2 | 6.5 | (0.2–36.0) | 3.1 | (1.0–7.2)* |
| Secondary and unspecific cancer | 41 | 19.3 | 2.1 | (1.5–2.9)** | 4 | 2.1 | 1.9 | (0.5–4.9) | 2.1 | (1.5–2.8)** |
| Haematopoietic and lymphatic system | | | | | | | | | | |
| Non-Hodgkin's disease | 22 | 19.1 | 1.2 | (0.7–1.7) | 1 | 1.8 | 0.6 | (0.0–3.1) | 1.1 | (0.7–1.7) |
| Multiple myelomatosis | 10 | 9.5 | 1.1 | (0.5–1.9) | 0 | 0.7 | 0.0 | (0.0–5.0) | 1.0 | (0.5–1.8) |
| Leukaemia | 17 | 24.1 | 0.7 | (0.4–1.1) | 1 | 1.8 | 0.6 | (0.0–3.1) | 0.7 | (0.4–1.1) |
| Other cancers | 26 | | | | 4 | | | | | |

*$P \leqslant 0.05$, **$P \leqslant 0.01$.

**Table II** Observed (O) and expected (E) numbers of cases and relative risk (RR) with 95% confidence interval (CI) of female breast cancer by age at diagnosis

| | *O* | *E* | *RR (CI)* |
|---|---|---|---|
| *Age at diagnosis* | | | |
| 1–44 years | 12 | 8.2 | 1.5 (0.8–2.6) |
| 45–54 years | 17 | 12.1 | 1.4 (0.8–2.3) |
| 55 + years | 12 | 11.8 | 1.0 (0.5–1.8) |
| Total | 41 | 32.0 | 1.3 (0.9–1.7) |

associations that are only marginally significant, or which have a wide confidence interval or are based on only a few observed cases, should be interpreted with caution.

The present study corroborates the associations between alcohol consumption and cancer risk of the upper digestive tract, the liver and the respiratory organs previously found in cohort studies (Sundby, 1967; Hakulinen *et al.*, 1974; Nicholls *et al.*, 1974; Monson & Lyon, 1975; Dean *et al.*, 1979; Hirayama, 1979; Jensen, 1979; Robinette *et al.*, 1979; Klatsky *et al.*, 1981; Schmidt & Popham, 1981; Gordon & Kannel, 1984; Pollack *et al.*, 1984; Kono *et al.*, 1986; Carstensen *et al.*, 1990). However, the relationship between alcoholism and cancers of the following sites in in disagreement with the literature.

### *Salivary glands and the lip*

In contrast to previous investigations, the present study showed increased morbidity due to cancer of the salivary glands in men: two developed cancer of the parotic gland, one cancer of the submandibular glands and four developed unspecified cancers of the salivary glands. This may reflect a direct action of the alcoholic beverage on the mucosa/glands. The decreased morbidity of lip cancer is probably because the cohort members were less likely to work outdoors and to be exposed to ultraviolet light than the Danish population as a whole. The lack of previous similar findings may be because of the smaller numbers of subjects studied. Also, the kind of alcohol ingested, together with other factors such as smoking, may be of relevance.

*Colon and rectum*

The relative risk of cancer of the colon or rectum was not increased. This is in line with the findings of several earlier investigations (Sundby, 1967; Hakulinen *et al.*, 1974; Nicholls *et al.*, 1974; Monson & Lyon, 1975; Graham *et al.*, 1978; Hirayama, 1979; Jensen, 1979, 1980; Robinette *et al.*, 1979; Klatsky *et al.*, 1981, 1988; Schmidt & Popham, 1981; Manousos *et al.*, 1983; Kono *et al.*, 1986; Potter & McMichael, 1986; Tuyns, 1988). Our study thus does not support the alleged association between alcoholic beverages (Wynder & Shigematsu, 1967; Pickle *et al.*, 1984; Hirayama, 1989; Carstensen *et al.*, 1990; Freudenheim *et al.*, 1990; Longnecker, 1990) including beer (Wynder & Shigematsu, 1967; Miller *et al.*, 1983; Pollack *et al.*, 1984; Kabat *et al.*, 1986; Kune *et al.*, 1987; Carstensen *et al.*, 1990; Freudenheim *et al.*, 1990) and the development of rectal cancer in particular. Owing to the size of the present study the relative risk estimate of 1.0 is quite precise, and it is unlikely that these heavy alcoholics have a more than 30% increased risk. Furthermore, a meta-analysis of 27 studies revealed no conclusive relationship (Longnecker *et al.*, 1990). The conflicting results may, in part, be explained by the differences in drinking habits of the study groups, and thus variations in displacement of cancer inhibitors from the diet by alcohol (Stemmermann *et al.*, 1990).

*Pancreas*

Our results suggested a marginally increased risk of pancreatic cancer. The association between pancreas cancer and smoking may be regarded as causal (IARC, 1986; Hiatt *et al.*, 1988), and smoking may be the confounder responsible for the increased risks found in our study and some other studies (Durbec *et al.*, 1983; Heuch *et al.*, 1983; Raymond *et al.*, 1987; Cuzick & Babiker, 1989; Carstensen *et al.*, 1990).

*Pleura*

Cancer of the pleura developed more often in the alcoholic men than expected (RR = 2.8; 95% CI 1.4–5.0). All but one of the 12 cases were histologically verified mesotheliomas. Associations between alcohol intake and mesothelioma have not been previously described.

*Breast*

Female breast cancer was slightly and non-significantly increased in our study, owing to an increased risk among the premenopausal (9–44 years) and menopausal (45–54 years) women. In the last decade three cohort studies (Hiatt & Bawol, 1984; Schatzkin *et al.*, 1987; Willet *et al.*, 1987) and several case–control studies have shown a similar tendency for an increased risk of breast cancer in women with even a very light alcohol intake, i.e. a drink or so daily, compared with abstainers (Lê *et al.*, 1984; Talamini *et al.*, 1984; La Vecchia *et al.*, 1985; Harvey *et al.*, 1987; O'Connell *et al.*, 1987; Rohan & McMichael, 1988). This association has not been consistent (Byers & Funch, 1982; Begg *et al.*, 1983; Paganini-Hill & Ross, 1983; Webster *et al.*, 1983; Meara *et al.*, 1989; Rosenberg *et al.*, 1990). However, meta-analysis of 16 studies revealed a significant trend in breast cancer risk with increase in light alcohol intake (Longnecker *et al.*, 1988).

Some of the case–control studies adjusted for several confounding factors such as social status, reproduction, obesity, family history, tobacco and dietary parameters. The presumably lower socioeconomic status among the alcoholic women of the present cohort may cause some negative confounding (Talamini *et al.*, 1984; O'Connell *et al.*, 1987). Our results suggest that the association must be more complex than a direct effect of alcohol alone.

*Female genital organs*

We found an increased risk of cervical cancer morbidity. In previous cohort studies of alcoholics the numbers of women included were too small to draw any conclusions about a relationship between alcoholism and cervical cancer mortality. A case–control study from Lesotho, South Africa, found increased risk associated with alcohol use after adjustment for tobacco (Martin & Hill, 1984). This may indicate the effect of confounding factors such as sexual practices or social (Brinton *et al.*, 1987) and nutritional factors (La Vecchia *et al.*, 1984; Harris *et al.*, 1986; Brock *et al.*, 1988).

*Male genital organs*

Cancer of the prostatic gland was increased among the male alcoholics. This association has not been described previously. The lack of consistence with results of previous cohort studies of cancer incidence may be due to a higher alcohol intake (Hakulinen *et al.*, 1974; Jensen, 1979; Carstensen *et al.*, 1990) in the present study, and to the low mortality of prostate cancer. The aetiology of prostatic cancer is uncertain. Several factors have been suggested, such as fat intake (Graham *et al.*, 1983; Ross *et al.*, 1987; Kolonel *et al.*, 1988; Mettlin *et al.*, 1989), increased body mass index (Talamini *et al.*, 1986) and overweight (Snowdon *et al.*, 1984). None of these studies have reported information about alcohol consumption. Two studies of prostatic cancer have dealt with alcohol intake. One Japanese follow-up study found no association between mortality and alcohol intake (Hirayama, 1979). The other study was not designed to draw conclusions about alcohol, because half of the control group consisted of persons with alcohol-related cancers (Newell *et al.*, 1989).

*Urinary tract*

The incidence of bladder cancer in women was higher than expected. The explanatory factor is probably smoking, since several case–control studies have found an increased risk of bladder cancer in alcohol drinkers that is reduced after adjustment for smoking (Morgan & Jain, 1974; Mommsen *et al.*, 1982; Thomas *et al.*, 1983). Surprisingly, the risk was not increased in men, although the increased RR of lung cancer suggests that smoking is more frequent than in the reference population.

The incidence of kidney cancer was slightly increased. Previous studies have not found this association (Hirayama, 1979; Jensen, 1979; Robinette *et al.*, 1979); this finding also demands further investigation of possible confounding factors.

*Skin*

We found a significantly decreased incidence of melanomas of the skin. Skin melanoma is causally related to intermittent sun exposure of untanned skin, and it is likely that the lower RR is explained by lower exposure compared with the reference population. Like previous studies of melanoma in Denmark (Jensen, 1979; Østerlind, 1990) the present investigation provides no support for the hypothesis of an association between alcohol intake and melanoma mediated through an influence on the melanocyte-stimulating hormone (Williams, 1976).

*Endocrine system*

Cancer of the endocrine system occurred more frequently than expected in the cohort. All the cases, except one thymoma, were histologically verified suprarenal cancers. No cases of thyroid cancer were found, although an association with alcohol consumption has been suggested (Williams, 1976). We would expect to observe an increased risk of thyroid cancer because of the large numbers of subjects studied, and must doubt the existence of an association.

*Conclusion*

The present study of alcoholics corroborates the associations between alcoholism and cancer of the upper digestive tract and the liver.

The study also suggests that there may be an association between alcoholism and prostatic cancer, but no association was found for colorectal cancer. Female breast cancer was only slightly increased in this first study of a sizeable number of alcoholic women.

The Danish Cancer Society, The Augustinus Foundation, The Hafnia Foundation, Winthertur-Borgen Legacy, Jacob and Olga Madsen Legacy, and Ib Henriksen Legacy are acknowledged for financial support to this study.

### References

BEGG, C.B., WALKER, A.K.M., WESSEN, B. & ZELEN, M. (1983). Alcohol consumption breast cancer (letter). *Lancet*, **i**, 293–294.

BRINTON, L.A., TASHIMA, K.T., LEHMAN, H.F., LEVIN, R.S., MALLIN, K., SAVITZ, D.A., STOLLEY, P.D. & FRAUMENI, J.R. (1987). Epidemiology of cervical cancer by cell type. *Cancer Res.*, **47**, 1706–1711.

BROCK, K.E., BERRY, J., MOCK, P.A., MACLENNAN, R., TRUSWELL, A.S. & BRINTON, L.A. (1988). Nutrients in diet and plasma and risk of *in situ* cervical cancer. *J. Natl Cancer Inst.*, **80**, 580–585.

BYERS, T. & FUNCH, D.P. (1982). Alcohol and breast cancer (letter). *Lancet*, **i**, 799–800.

CARSTENSEN, J.M., BYGREN, L.P. & HATSCHEK, T. (1990). Cancer incidence among Swedish brewery workers. *Int. J. Cancer*, **45**, 393–396.

COLEMAN, M., DOUGLAS, A., HERMON, C. & PETO, J. (1986). Cohort study analysis with a fortran computer program. *Int. J. Epidemiol.*, **15**, 134–137.

COPENHAGEN MUNICIPAL OUT-PATIENT CLINIC FOR TREATMENT OF ALCOHOLISM (1960–80). Forchhammersvej 18, DK-1920 Copenhagen, Denmark. Annual Reports to the municipality. Originals in Danish.

CUZICK, J. & BABIKER, A.G. (1989). Pancreatic cancer, alcohol, diabetes mellitus, and gallbladder disease. *Int. J. Cancer*, **43**, 415–421.

DEAN, G., MACLENNAN, R., MCLOUGHLIN, H. & SHELLEY, E. (1979). Causes of death of blue-collar workers at a Dublin brewery. *Br. J. Cancer*, **40**, 581–589.

DURBEC, J.P., CHEVILLOTTE, G., BIDART, J.M., BERTHEZENE, P. & SARLES, H. (1983). Diet, alcohol, tobacco, and risk of cancer of the pancreas: a case–control study. *Br. J. Cancer*, **47**, 463–470.

FREUDENHEIM, J.L., GRAHAM, S., MARSHALL, J.R., HAUGHEY, B.P. & WILKINSON, G. (1990). Lifetime alcohol intake and risk of rectal cancer in Western New York. *Nutr. Cancer*, **13**, 101–109.

GORDON, T. & KANNEL, W.B. (1984). Drinking and mortality: the Framingham study. *Am. J. Epidemiol.*, **120**, 97–107.

GRAHAM, S., DAYAL, H., SWANSON, M., MITTELMAN, A. & WILKINSON, G. (1978). Diet in the epidemiology of cancer of the colon and rectum. *J. Natl Cancer Inst.*, **61**, 709–714.

GRAHAM, S., HAUGHEY, B., MARSHALL, J., PRIORE, R., BYERS, T., RZEPKA, T. & METTLIN, C. (1983). Diet in the epidemiology of carcinoma of the prostate gland. *J. Natl Cancer Inst.*, **70**, 687–692.

HAKULINEN, T., LEHTIMÄKI, L., LEHTONEN, M. & TEPPO, L. (1974). Cancer morbidity among two male cohorts with increased alcohol consumption in Finland. *J. Natl Cancer Inst.*, **52**, 1711–1714.

HARRIS, R.C.V., FORMAN, D., DOLL, R., VESSEY, M.P. & WALD, N.J. (1986). Cancer of the cervix uteri and vitamin A. *Br. J. Cancer*, **53**, 653–659.

HARVEY, E.B., SCHAIRER, M.S., BRINTON, L.A., HOOVER, R.N. & FRAUMENI, J.F. (1987). Alcohol consumption and breast cancer. *J. Natl Cancer Inst.*, **78**, 657–661.

HEUCH, I., KVOLE, G., JACOBSEN, B.K. & BJELKE, E. (1983). Use of alcohol, tobacco and coffee, and risk of pancreatic cancer. *Br. J. Cancer*, **48**, 637–643.

HIATT, R.A. & BAWOL, R.D. (1984). Alcoholic beverage consumption and breast cancer incidence. *Am. J. Epidemiol.*, **120**, 676–683.

HIATT, R.A., KLATSKY, A.L. & ARMSTRONG, M.A. (1988). Pancreatic cancer, blood glucose, and beverage consumption. *Int. J. Cancer*, **41**, 794–797.

HIRAYAMA, T. (1979). Diet and cancer. *Nutr. Cancer*, 67–81.

HIRAYAMA, T. (1989). Association between alcohol consumption and cancer of the sigmoid colon: observations from a Japanese cohort study. *Lancet*, **ii**, 725–727.

IARC (1986). IARC Monographs on the evaluation of carcenogenic risks of chemicals to humans. *Tobacco smoking*. Vol. 38. IARC: Lyon.

IARC (1988). IARC Monographs on the evaluation of carcinogenic risks to humans. *Alcohol drinking*. Vol. 44. IARC: Lyon.

JENSEN, E. & VANG, H. (1983). Treatment of alcoholics with antabuse (disulfiram) under different circumstances (abstract). *The 29th International Institute on the Prevention and Treatment of Alcoholism, Zagreb, Yugoslavia, 27th June – 1st July.*

JENSEN, O.M. (1979). Cancer morbidity and causes of death among Danish brewery workers. *Int. J. Cancer*, **23**, 454–463.

JENSEN, O.M. (1980). Cancer morbidity and causes of death among Danish brewery workers. IARC: Lyon.

KABAT, G.C., HOWSON, C.P. & WYNDER, E.L. (1986). Beer consumption and rectal cancer. *Int. J. Epidemiol.*, **15**, 494–501.

KLATSKY, A.L., FRIEDMAN, G.D. & SIEGELAUB, A.B. (1981). Alcohol and mortality: a ten-year Kaiser-Permanente experience. *Ann. Intern. Med.*, **95**, 139–145.

KLATSKY, A.L., ARMSTRONG, M.A., FRIEDMAN, G.D. & HIATT, R.A. (1988). The relations of alcoholic beverage use to colon and rectal cancer. *Am. J. Epidemiol.*, **128**, 1007–1015.

KOLONEL, L.N., YOSHIZAWA, C.N. & HANKIN, J.H. (1988). Diet and prostatic cancer: a case–control study in Hawaii. *Am. J. Epidemiol.*, **127**, 999–1012.

KONO, S., IKEDA, M., TOKUDOME, S., NISHIZUMI, M. & KARATSUNE, M. (1986). Alcohol and mortality: a cohort study of male Japanese Physicians. *Int. J. Epidemiol.*, **15**, 527–532.

KUNE, S., KUNE, G.A. & WATSON, L.F. (1987). Case–control study of alcoholic beverages as etiologic factors: the Melbourne colorectal cancer study. *Nutr. Cancer*, **9**, 43–56.

LA VECCHIA, D., FRANCESCHI, S., DECARLI, A., GENTILE, A., FASOLI, M., PAMPALLONA, S. & TOGNONI, G. (1984). Dietary vitamin A and the risk of invasive cervical cancer. *Int. J. Cancer*, **34**, 319–322.

LA VECCHIA, D., DECARLI, A., FRANCESCHI, S., PAMPALLONA, S. & TOGNONI, G. (1985). Alcohol consumption and the risk of breast cancer in women. *J. Natl Cancer Inst.*, **75**, 61–65.

LE, M.G., HILL, C., KRAMAR, A. & FLAMANT, R. (1984). Alcoholic beverage consumption and breast cancer in a French case–control study. *Am. J. Epidemiol.*, **120**, 350–357.

LONGNECKER, M.P. (1990). A case–control study of alcoholic beverage consumption in relation to risk of cancer of the right colon and rectum in men. *Cancer Caus. Contr.*, **1**, 5–14.

LONGNECKER, M.P., BERLIN, J.A., ORZA, M.J. & CHALMERS, T.C. (1988). A meta-analysis of alcohol consumption in relation to the risk of breast cancer. *JAMA*, **260**, 652–656.

LONGNECKER, M.P., ORZA, M.J., ADAMS, M.E., VIOQUE, J. & CHALMERS, T.C. (1990). A meta-analysis of alcoholic beverage consumption in relation to risk of colorectal cancer. *Cancer Caus. Contr.*, **1**, 59–68.

MANOUSOS, O., DAY, N.E., TRICHOPOULOS, D., GEROVASSILIS, F. & TZONOU, A. (1983). Diet and colorectal cancer: a case–control study in Greece. *Int. J. Cancer*, **32**, 1–5.

MARTIN, P.M.D. & HILL, G.B. (1984). Cervical cancer in relation to tobacco and alcohol consumption in Lesotho, Southern Africa. *Cancer Detect. Prev.*, **7**, 109–115.

MEARA, J., MCPERSON, K., ROBERTS, M., JONES, L. & VESSEY, M. (1989). Alcohol, cigarette smoking, and breast cancer. *Br. J. Cancer*, **60**, 70–73.

METTLIN, C., SELENSKAS, S., NATARAJAN, N. & HUBEN, R. (1989). Beta-carotene and animal fats and their relationship to prostate cancer risk. A case–control study. *Cancer*, **64**, 605–612.

MILLER, A.B., HOWE, G.R., JAIN, M., CRAIB, K.J.B. & HARRISON, L. (1983). Food items and food groups as risk factors in a case–control study of diet and colorectal cancer. *Int. J. Cancer*, **32**, 155–161.

MOMMSEN, S., AAGAARD, J. & SELL, A. (1982). An epidemiological case–control study of bladder cancer in males from a predominantly rural district. *Eur. J. Cancer Clin. Oncol.*, **18**, 1205–1210.

MONSON, R.R. & LYON, J.L. (1975). Proportional mortality among alcoholics. *Cancer*, **36**, 1077–1079.

MORGAN, R.W. & JAIN, M.G. (1974). Bladder cancer: smoking, beverages, and artificial sweeteners. *Can. Med. Assoc.*, **111**, 1067–1070.

NEWELL, G.R., FUEGER, J.J., SPITZ, M.R. & BABAIAN, R.J. (1989). A case–control study of prostate cancer. *Am. J. Epidemiol.*, **130**, 395–398.

NICHOLLS, P., EDWARDS, G. & KYLE, E. (1974). Alcoholics admitted to four hospitals in England. II. General and cause-specific mortality. *Q. J. Study Alcohol*, **35**, 841–855.

O'CONNELL, D.L., HULKA, B.S., CHAMPLESS, L.E., WILKINSON, W.E. & DEUBNER, D.C. (1987). Cigarette smoking, alcohol consumption, and breast cancer risk. *J. Natl Cancer Inst.*, **78**, 229–234.

ØSTERLIND, A. (1990). Malignant melanoma in Denmark. *Acta Oncol.*, **29**, 1–22.

ØSTERLIND, A. & JENSEN, O.M. (1985). Evaulation of registration of cancer cases in Denmark in 1977. Preliminary evaluation of registration of cancer cases by the Cancer Registry and the National Patient Registry. *Ugeskr. Laeger*, **147**, 2483–2488.

PAGANINI-HILL, A. & ROSS, R.K. (1983). Breast cancer and alcohol consumption (letter). *Lancet*, **ii**, 626–627.

PICKLE, L.W., GREEN, M.H., ZIEGLER, R.G., TOLEDO, A. & HOOVER, R. (1984). Colorectal cancer in Rural Nebraska. *Cancer Res.*, **44**, 363–369.

POLLACK, E.S., NOMURA, A.M.Y., HEILBRUN, L.K., STEMMERMANN, G.N. & GREEN, S.B. (1984). Prospective study of alcohol consumption and cancer. *N. Engl. J. Med.*, **310**, 617–621.

POTTER, J.D. & MCMICHAEL, A.J. (1986). Diet and cancer of the colon and rectum: a case–control study. *J. Natl Cancer Inst.*, **76**, 557–569.

RAYMOND, L., INFANTE, F., TUYNS, A.J., VOIROL, M. & LOWENFELS, A.B. (1987). Diet and pancreatic cancer. *Gastroenterol. Clin. Biol.*, **11**, 488–492.

ROBINETTE, C.D., HRUBEC, Z. & FRAUMENI Jr, J.F. (1979). Chronic alcoholism and subsequent mortality in World War II veterans. *Am. J. Epidemiol.*, **109**, 687–700.

ROHAN, T.E. & MCMICHAEL, A.J. (1988). Alcohol consumption and risk of breast cancer. *Int. J. Cancer*, **41**, 695–699.

ROSENBERG, L., PALMER, J.R., MILLER, D.R., CLARKE, E.A. & SHAPIRO, S. (1990). A case–control study of alcoholic beverage consumption and breast cancer. *Am. J. Epidemiol.*, **131**, 6–14.

ROSS, R.K., SHIMZU, H., PAGANINI-HILL, A., HONDA, G. & HENDERSON, B.E. (1987). Case–control studies of prostate cancer in blacks and whites in Southern California. *J. Natl Cancer Inst.*, **78**, 869–874.

ROTHMAN, K.J. & BOICE Jr, J.D. (1982). *Epidemiologic Analysis with a Programmable Calculator*, 2nd edn. Epidemiologic Resources: Boston.

SCHATZKIN, A., JONES, D.Y., HOOVER, R.N., TAYLOR, P.R., BRINTON, L.A., ZIEGLER, R.G., HARVEY, E.B., CARTER, C.L., LICITRA, L.M., DUFOUR, M.C. & LARSON, D.B. (1987). Alcohol consumption and breast cancer in the epidemiologic follow-up study of First National Health and Nutrition Examination Survey. *N. Engl. J. Med.*, **316**, 1169–1173.

SCHMIDT, W. & POPHAM, R.E. (1981). The role of drinking and smoking in mortality from cancer and other causes in male alcoholics. *Cancer*, **47**, 1031–1041.

SNOWDON, D.A., PHILLIPS, R.L. & CHOI, W. (1984). Diet, obesity, and risk of fatal prostate cancer. *Am. J. Epidemiol.*, **120**, 244–250.

STATISTICAL YEARBOOKS OF DENMARK (1955, 1988). The Statistical Department: Copenhagen.

STEMMERMANN, G.N., MONURA, A.M.Y., CHYOU, P.-H. & YOSHIZAWA, C. (1990). Prospective study of alcohol intake and large bowel cancer. *Dig. Dis. Sci.*, **35**, 1414–1420.

STORM, H.H. (1984). Validity of death certificates for cancer patients in Denmark 1977. Danish Cancer Registry, Danish Cancer Society (original in Danish): Copenhagen.

STORM, H.H., MANDERS, T., SPRØGEL, P., BANG, S. & JENSEN, O.M. (1990). Cancer incidence in Denmark 1987. Danish Cancer Society, Danish Cancer Registry: Copenhagen.

SUNDBY, P. (1967). *Alcoholism and Mortality*, thesis, University of Oslo. Universitetsforlaget: Oslo.

TALAMINI, R., LA VECCHIA, D., DECARLI, A., FRANCESCHI, S., GRATTONI, E., GRIGOLETTO, E., LIBERATI, A. & TOGNONI, G. (1984). Social factors, diet, and breast cancer in a Northern Italian population. *Br. J. Cancer*, **49**, 723–729.

TALAMINI, R., LA VECCHIA, D., DECARLI, A., NEGRI, E. & FRANCESCHI, S. (1986). Nutrition, social factors, and prostatic cancer in a Northern Italian population. *Br. J. Cancer*, **53**, 817–821.

THOMAS, D.B., UHL, C.N. & HARTGE, P. (1983). Bladder cancer and alcoholic beverage consumption. *Am. J. Epidemiol.*, **118**, 720–727.

TUYNS, A.J. (1988). Beer consumption and rectal cancer. *Rev. Epidém. Santé Publ.*, **36**, 144–145.

WEBSTER, L.A., LAYDE, P.M., WINGO, P.A. & ORY, H.W. (1983). Alcohol consumption and risk of breast cancer. Lancet, **ii**, 724–726.

WILLET, W.C., STAMPFER, M.J., COLDITZ, G.A., ROSNER, B.A., HENNEKENS, C.H. & SPEIZER, F.E. (1987). Moderate alcohol consumption and the risk of breast cancer. *N. Engl. J. Med.*, **316**, 1174–1180.

WILLIAMS, R.R. (1976). Breast and thyroid cancer and malignant melanoma promoted by alcohol-induced pituitary secretion of prolactin, TSH, and MSH. *Lancet*, **i**, 996–999.

WYNDER, E.L. & SHIGEMATSU, T. (1967). Environmental factors of cancer of colon and rectum. *Cancer*, **20**, 1520–1561.